# Unexpected Diversity of Xenoscelinae in Priabonian European Amber: The Third Xenosceline Species from Rovno Amber

**DOI:** 10.3390/life13030636

**Published:** 2023-02-24

**Authors:** Georgy Yu. Lyubarsky, Evgeny E. Perkovsky, Dmitry V. Vasilenko

**Affiliations:** 1 Zoological Museum, Moscow Lomonosov State University, Bol’shya Nikitskaya 2, Moscow 125009, Russia; 2 I. I. Schmalhausen Institute of Zoology, National Academy of Sciences of Ukraine, Bogdan Khmielnitsky Str. 15, 01030 Kiev, Ukraine; 3 Natural History Museum of Denmark, Universitetsparken 15, 2100 Copenhagen, Denmark; 4 A.A. Borissiak Paleontological Institute, Russian Academy of Sciences, Moscow 117647, Russia; 5 Paleontological Laboratory, Cherepovets State University, Cherepovets 162600, Russia

**Keywords:** *Xenophagus*, taxonomy, late Eocene, Baltic amber, Bitterfeld amber, sympatry

## Abstract

*Xenophagus simutniki* sp. n. is described from a late Eocene Rovno amber specimen. The new species is similar to the fossil *Xenophagus popovi* Lyubarsky et Perkovsky, 2017 from the late Eocene Baltic amber (W Russia), differing in the medially notched anterior margin of the pronotum. The Rovno xenosceline fauna is the richest among both extant and extinct faunas. This fauna includes the extinct genera *Xenophagus* Lyubarsky & Perkovsky, 2017 and *Xenohimatium* Lyubarsky & Perkovsky, 2012, which are closest to the extant Mediterranean *Xenoscelis* Wollaston 1864 and the representative of the extant boreal genus *Zavaljus* Reitter, 1880. A key to extinct species of the subfamily Xenoscelinae is presented. The possible reasons of xenoscelines abundance in European amber forests are discussed.

## 1. Introduction

Erotylidae, pleasing fungus beetles and lizard beetles, are distributed worldwide and include approximately 3200 species in 300 genera and six subfamilies [1,2]. The classification of the group was most recently revised by Leschen [2], who combined the families Languriidae and Erotylidae based on a parsimony analysis [1,3,4]. One of the major problems remaining with the classification of erotylids is the paraphyly of Xenoscelinae and Loberinae (details are discussed in Leschen [2]). The family contains phytophagous, mycophagous, and saprophagous species, and a few other taxa that feed on pollen and dead wood. Most species are free living, but–as noted by Leschen and Buckley [5]—*Chasmatodera* Arrow, 1943 and *Bancous* Pic, 1946 (Erotylinae from Afrotropical and Oriental region) are known from nests of the fungus-cultivating termites [6], and wingless species of *Loberopsyllus* Martinez & Barrera, 1966 (Cryptophilinae from southern Mexico and Costa Rica) occur phoretic on neotomine rodents [7]. *Lepidotoramus* Leschen, 1997 (Cryptophilini from Amazon Basin) may be endoparasitic on Lepidoptera pupae [8]. The data mainly refer to the biology of adult beetles; there is much less information on the development of larvae.

Several extant genera have been recently erected in the subfamily Xenoscelinae with the list given in Lyubarsky and Perkovsky [9]. In last the last few years, four new extinct Xenoscelinae genera have been described (*Microzavaljus* Lyubarsky & Perkovsky, 2018; *Warnis* Lyubarsky, Perkovsky & Alekseev, 2016; *Xenophagus* Lyubarsky & Perkovsky, 2017; *Xenohimatium* Lyubarsky & Perkovsky, 2012) resulting in a total of 12.

Several Erotylidae fossils are known from the late Eocene amber faunas: erotyline *Triplax contienensis* Alekseev, 2014, languriine *Serramorphus rasnitsyni* Lyubarsky & Perkovsky, 2017, and xenosceline *Microzavaljus saxonicum* Lyubarsky & Perkovsky, 2018 in Bitterfeld amber [9,10,11], languriine *Thallisellites olgae* Kupryjanowicz, Lyubarsky & Perkovsky, 2021 in Baltic amber, *Cycadophila mumia* Alekseev, 2017 (Pharaxonothinae), xenoscelines *Warnis tvanksticus* Lyubarsky, Perkovsky & Alekseev, 2016 and *Xenophagus popovi* Lyubarsky & Perkovsky, 2017 in Baltic amber [12,13,14,15], xenoscelines *Xenohimatium rovnense* Lyubarsky & Perkovsky, 2012 [16], the new species of *Xenophagus* Lyubarsky & Perkovsky, 2017 described in the present paper, and *Zavaljus lyubarskyi* Alekseev & Bukejs, 2022 [17] in Rovno amber.

Rovno amber is the southern coeval of the famous Baltic amber from Rovno, Kiev (western part), Volyn and Zhitomir regions of Ukraine [18,19,20] and adjacent districts of Belarus [21]. Rovno amber biota was reviewed in [22,23,24], [25] and references therein). More than 70 beetle species from 35 families [17,19,26,27,28,29,30,31,32,33,34,35,36,37,38,39,40,41,42,43,44,45,46,47,48,49,50,51,52,53] are reported; less than 15% of Rovno beetle species are known from Baltic amber [53,54,55] and references therein.

The purpose of this work was to describe the third erotylid species from Rovno amber and analyze the reasons for the relict nature of the modern distribution of the tribe.

## 2. Material and Methods

The new Rovno amber pleasing fungus beetle specimen was purchased from an amber dealer, who obtained the amber mostly in the Varash district of the Rovno region, the most important source of the new Rovno amber taxa in the last few years [30,32,33,38,40,41,45,50,51,52,56,57,58,59]. It was included in the clear piece of amber, weighting 7.6 g after the primary treatment. The specimen was cut and polished by Anatoly P. Vlaskin (SIZK) for the best visibility of the fossil to the subrectangular piece 10 × 6.5 × 1.6 mm.

Morphological terminology for the body parts of the beetles is following the work of Leschen [2].

Photographs were taken at the Schmalhausen Institute of Zoology, National Academy of Sciences of Ukraine (Kiev, SIZK) using the microscope Leica Z16 APO stereomicroscope equipped with a Leica DFC 450 camera. Dimensions were measured by 10 × 23 Leica 10450023Widefield Adjustable Eyepiece on Leica MZ APO.

Eleven extant specimens cited in the text were collected by late Alexey Yu. Isaev (Ulyanovsk State University, Russia) and are deposited in the Zoological Museum of Lomonosov State University (Moscow, ZMSU).

The holotype is deposited in collection of Schmalhausen Institute of Zoology.

## 3. Results

### Systematic Paleontology

Order Coleoptera Linnaeus, 1758
Superfamily Cucujoidea Latreille, 1802
Family Erotylidae Latreille, 1802
Subfamily Xenoscelinae Ganglbauer, 1899
Genus *Xenophagus* Lyubarsky & Perkovsky, 2017
Type species: *Xenophagus popovi* Lyubarsky & Perkovsky, 2017
Species composition. Type species, *Xenophagus simutniki* sp.n.

**Figure 1:**
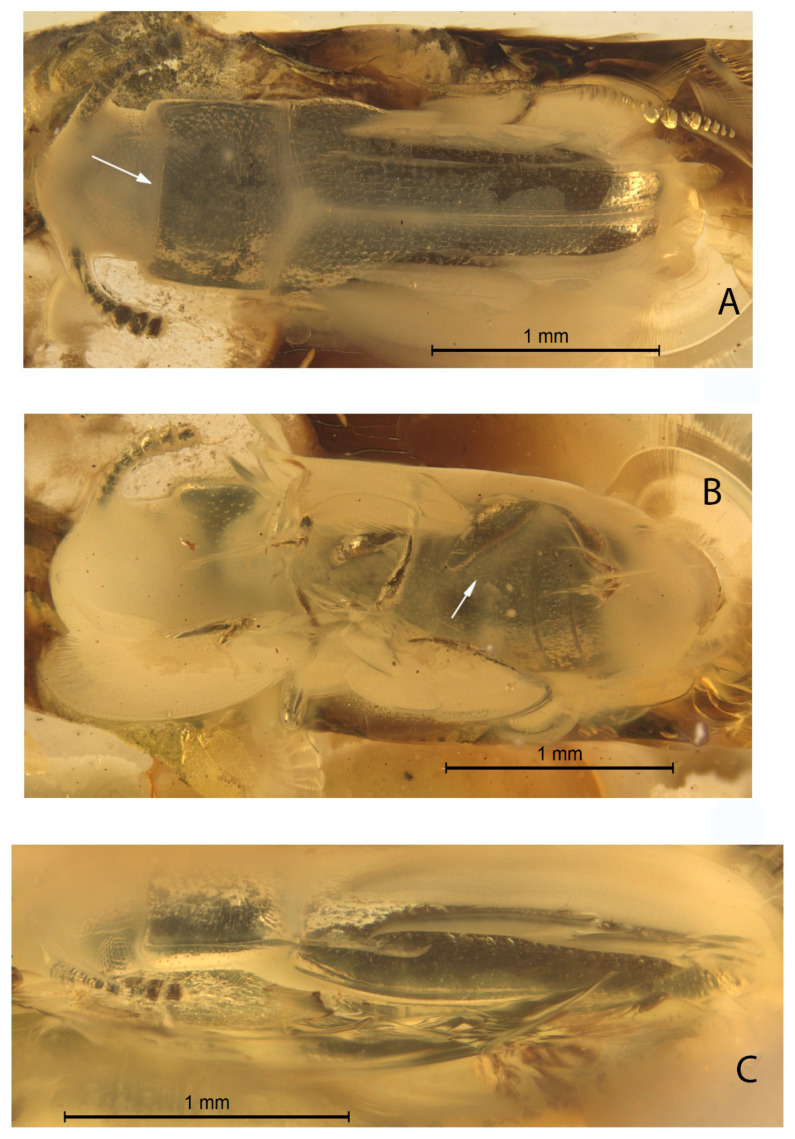
Holotype *Xenophagus simutniki* sp.n., images: (**A**)—dorsal view, arrow indicates notch of pronotum; (**B**)—ventral view, arrow indicates submetacoxal line; (**C**)—left lateral view.

**Figure 2:**
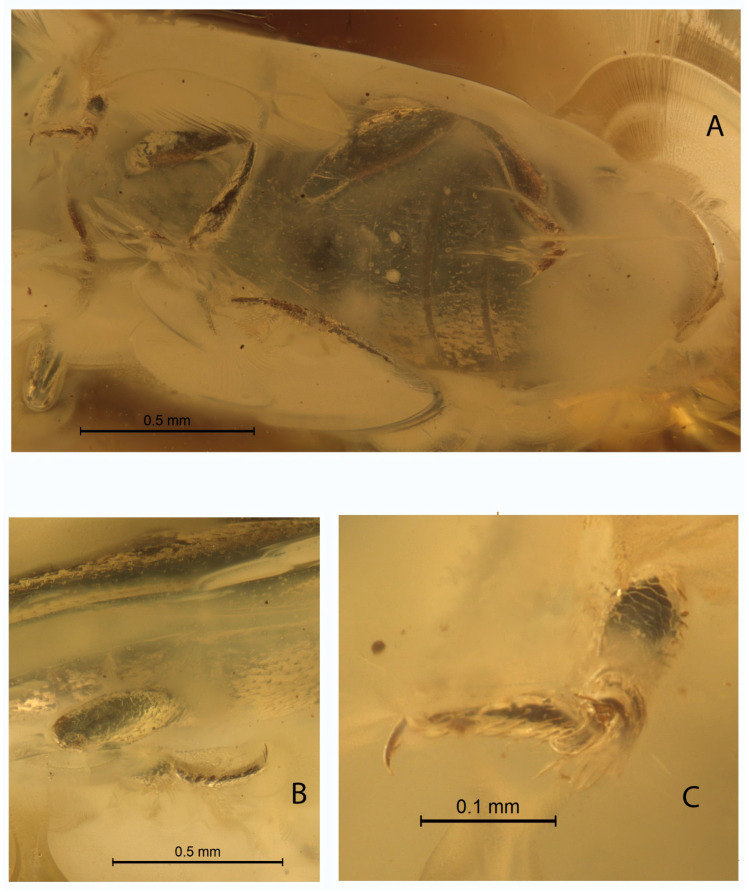
Holotype *Xenophagus simutniki* sp.n., images: (**A**)—ventral view of abdomen with submesocoxal lines; (**B**)—lateral view of abdomen with metatarsus; (**C**)—metatarsus.

**Figure 3:**
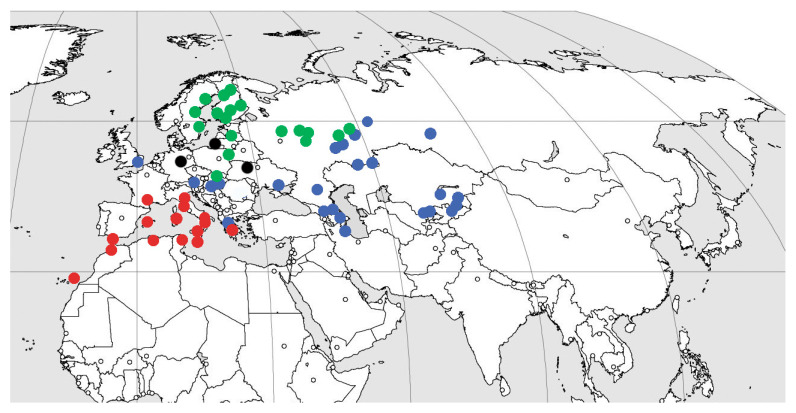
Distribution of Palearctic xenosceline genera. Green dots—*Zavaljus*; blue dots—*Macrophagus*; red dots—*Xenoscelis*; black dots—Priabonian amber deposits: Baltic, Rovno, and Bitterfeld amber.

**Figure 4:**
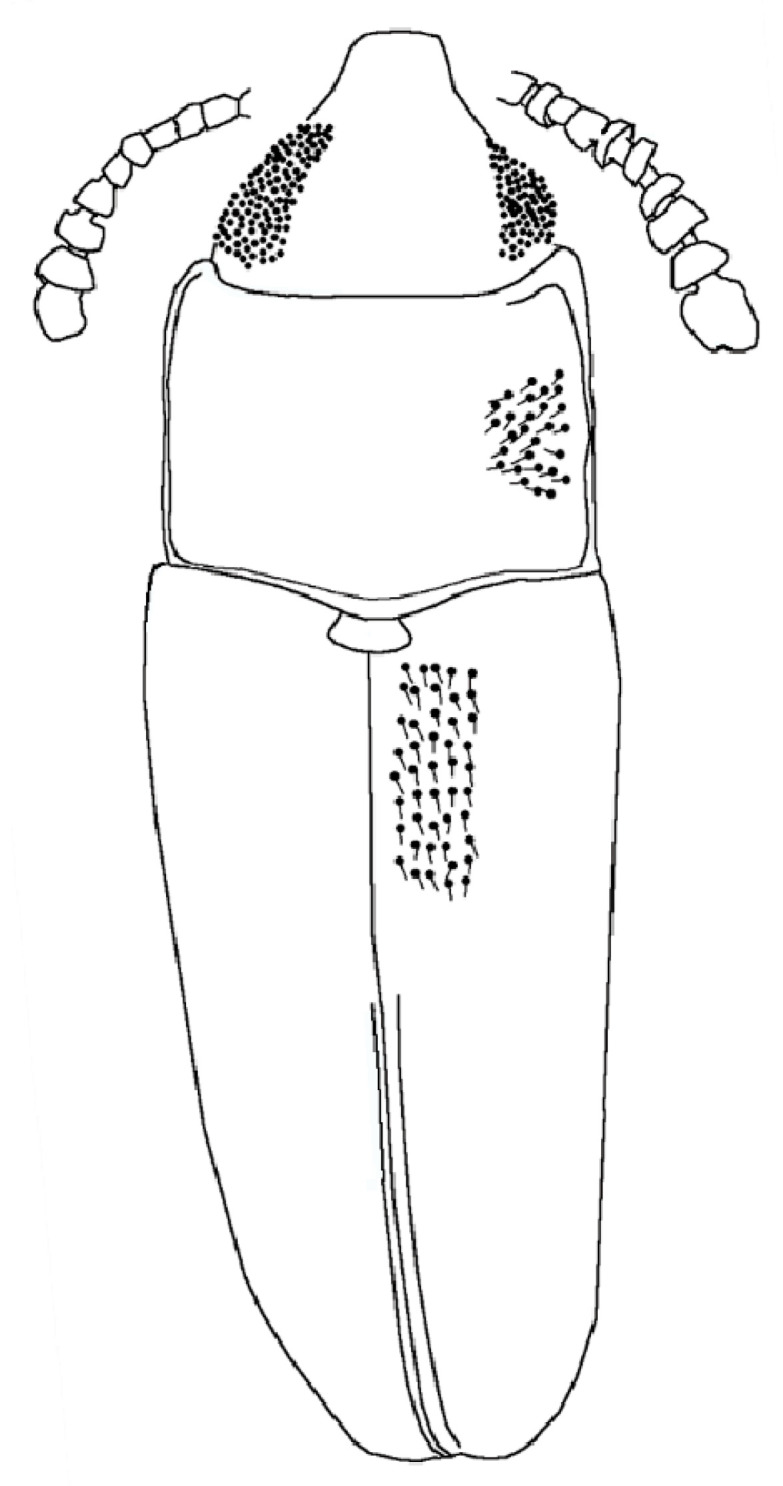
*Xenophagus simutniki* sp.n., drawing, dorsal view.

*Xenophagus simutniki* Lyubarsky & Perkovsky, sp.n.
Figure 1, Figure 2, Figure 3 and Figure 4.
http://zoobank.org/urn:lsid:zoobank.org:act:97E43669-56D8-4103-BC4E-D273399E242A (accessed on 20 February 2023)
**Etymology.** Named in honor of our friend, hymenopterist Dr. Sergej A. Simutnik.
MATERIAL. Holotype: SIZK UA-28134, Rovno amber, late Eocene.
Diagnosis. Anterior margin of the pronotum with the median notch (Figure 1A and Figure 4); antennae comparatively short, not extending beyond posterior margin of pronotum when directed posteriad (Figure 1A–C); abdominal ventrite 1 twice as long as ventrite 2 (Figure 2A).
DESCRIPTION. Body parallel-sided, 2.5 times longer than wide, dorsal vestiture short and decumbent (Figure 1A), dorsum weakly convex. Body length 2.7 mm, width 0.9 mm.

Head width equals 0.8 of the pronotal width. Compound eye hemispherical, comparatively large. Facets moderate in size, approximately equal to diameter of dorsal head puncture. Dorsal punctuation of head: punctures medium in size, distance between neighboring punctures equal to 1.5 diameter of puncture. Antennae 11-merous with three-segmented club, comparatively short, not extending beyond posterior margin of pronotum when directed posteriad. Antennal club slightly flattened. Antennomeres 9–10 transverse (Figure 1A). Antennomeres 3–5 very slightly elongate, 6th antennomere as long as wide, 7th and 8th antennomeres slightly transverse. Terminal antennomere as long as wide, with rounded tip. Antennomeres 3–7 subequal in length. Antennal furrows not present.

Pronotum parallel-sided, without lateral callosity and denticles, pronotal length 0.7 times the width, about 0.4 the elytral length. Anterior margin with median notch (Figure 1A). Lateral pronotal margin slightly flattened at anterolateral angles. Lateral margins and base of pronotum bordered. Base of pronotum with shallow, transverse depression; basal pits not present, basal furrow not present (Figure 1C). Posterior pronotal margin with basal lobe. Pronotum strongly and densely punctured, intervening spaces about 1.5 times as wide as puncture diameters. Posterolateral angles rectangular.

Intercoxal distance between mesocoxae 1.5 times shorter than width of mesocoxa. Intercoxal distance between metacoxae greater than diameter of metacoxa. Pre-, meso-, and metasternum strongly punctured. Legs slender, tibia slightly dilated apically. Tibia with bristle-like “crown” at apex, with short terminal spurs. Tarsomeres elongated, not lobate (Figure 2B,C). Tarsomeres 1–4 about same length, fifth tarsomere the longest, nearly as long as combined length of remaining tarsomeres (Figure 2B). Claw without notches, smooth, about 1/4 length of tarsomere 5.

Scutellar shield transverse, 2.5 times as long as wide. Elytral length 1.9 times as long as wide. Elytra narrowed and rounded apically. Elytra strongly punctured, intervening spaces about twice as wide as puncture diameters. Much smaller, secondary punctures are visible on the intervening spaces. Elytral surface not shagreened. Lateral margin of elytra is weakly distinguishable from above because of the boundaries between the layers of amber.

Abdominal ventrite 1 twice as long as ventrite 2 (Figure 2A). Submetacoxal lines present (Figure 2A). Abdominal punctures with bristles.

**Key to extinct species of subfamily Xenoscelinae**

1. Submetacoxal lines present (Figure 1B and Figure 2A)………………………………………….2
— Submetacoxal lines not present……………………………………………………………….……4
2. Elytral punctures irregular. .…………………………………………………………………………3
— Elytral punctures arranged in rows. Baltic amber ………… *Warnis tvanksticus* Lyubarsky, Perkovsky & Alekseev, 2016
3. Anterior margin of pronotum notched (Figure 1A). Rovno amber..……*Xenophagus simutniki* Lyubarsky & Perkovsky, sp. n.
— Anterior margin of pronotum without notch. Baltic amber …………*Xenophagus popovi* Lyubarsky & Perkovsky, 2017
4. Pronotum widest across apical one third. ..……………………………………………………5
— Pronotum almost completely parallel-sided. Rovno amber..………… *Xenohimatium rovnense* Lyubarsky & Perkovsky, 2012
5. Antenna long, extending beyond posterior edge of pronotum when directed posteriad. Rovno amber ..………*Zavaljus lyubarskyi* Alekseev & Bukeis, 2022
— Antenna short, extending towards midlength of pronotum when directed posteriad. Bitterfeld amber ………*Microzavaljus saxonicus* Lyubarsky & Perkovsky, 2018

## 4. Discussion

Hitherto, no erotylid genera were known simultaneously from all three studied late Eocene European amber faunas. In that respect, this family differs, for instance, from cryptophagids, where all yet known Rovno (and Bitterfeld) amber genera are extant and known from the Baltic amber as well [54].

Subfamily Xenoscelinae comprises the following extant genera: New Zealand: *Loberonotha* Sen Gupta & Crowson, 1969 (one species); Neotropical: *Othniocryptus* Sharp, 1900 (one species; Panama, South America); Australian: *Protoloberus* Leschen, 2003 (one species; Queensland, New South Wales); Afrotropical: *Arrowcryptus* Leschen & Wegrzynowicz, 2008 (two species, tropical Africa), *Xenocryptus* Arrow, 1929 (two species; South Africa and Western Australia); Palaearctic (Figure 3): *Xenoscelis* Wollaston, 1864 (Mediterranean, one species); *Zavaljus* Reitter, 1880 (one extant species from Northern and north of Eastern Europe) and *Macrophagus* Motschulsky, 1845 (one species; from Austria, Hungary and Slovakia to Gilan and Kyrgyzstan). Xenoscelinae also includes four extinct genera (*Xenohimatium* from Rovno amber, *Warnis* from Baltic amber, *Xenophagus* from Baltic and Rovno amber, and *Microzavaljus* from Bitterfeld amber).

In each region and each amber Lagerstätte, one–two species of each genus are present. Three genera and three species that occur sympatrically in Rovno amber is the maximum yet observed local diversity for this subfamily, while the six species from European amber already represent 60% of the extant global diversity of the group and 71% of the genus-rank one.

The highest present-day diversity of the subfamily (three species in three genera) is recorded in Western Palaearctic. Yet even there, no distribution area overlapping is observed for different genera and species. Even in the eastern part of European Russia with the closest recorded distance between the populations of *Zavaljus* and *Macrophagus*, their nearest known localities are at least 150 km distant (Ulyanovsk Region, our data: *Zavaljus* from Palatovo village, Valgusskoye rural settlement, Inza District, under alder bark; *Macrophagus* from chalk steppe: Arskoye village, Ulyanovsk city district, 12 August 1990; Zykovo village, Novospasskoye urban settlement, 23 August 1990; Akulovka, Nikolaevsky District, 7 June 1990 and steppe (“mowing”): Shilovka village, Tushninskoye rural settlement, Sengileevsky District, 15 June 1987). This can be explained by the different ecological preferences of the mentioned taxa in present-day environments, i.e., dead wood association for *Zavaljus brunneus* (Gyllenhal, 1808) [60].

In contrast, adults of *Xenoscelis deplanatus* (Wollaston, 1862) are known to occur in dead stems of the *Euphorbia* shrubs, under decaying vegetation and under stones (especially in winter). It should be noted that *Macrophagus* is anthophilous, collected from bee nests (*Anthophora* Latreille 1803 and *Halictus* Latreille, 1804), and *Zavaljus* is more associated with wasps [61].

On the contrary, in European late Eocene amber faunas xenosceline genera obviously coexisted. Equally in contrast with the present times, Xenoscelinae dominated clearly in the late Eocene amber faunas of Europe, comprising a third of all known Erotylidae in Bitterfeld, half in Baltic, and being the only erotylid subfamily known from Rovno amber. In other words, xenoscelines encompassed half of all Erotylidae at the northern coast of Subparatethys and two thirds of them at its southern coast.

While the area of Russoscandia Island was comparable with Greenland, and the allopatry of the Baltic xenoscelines is theoretically possible, Volyn land with Rovno amber forest was rather small [62], so the xenoscelines sympatry was practically inevitable.

Extant fauna of xenoscelines includes three tropical species (33%) from the genera *Othniocryptus* and *Arrowcryptus*, two (22%) species from temperate regions (Figure 3), but the largest diversity of species—four (44%) are known from the subtropics, except for *Xenoscelis*, which is distributed all through the Southern Hemisphere. It is natural to assume that increasing seasonality of European post-Eocene climate led to a sharp decline in the abundance and diversity of xenoscelines, as well as to their transition to allopatric distribution. The transition to allopatric distribution was likely driven by narrowing and decrease in available ecological niches. Xenoscelines, adapted to equable climate, were likely quite preadapted for the areas with a Mediterranean climate, and part of them moved further south (genera *Xenophagus* and *Xenohimatium* most close to the extant Mediterranean genus *Xenoscelis* Wollaston, 1864; of course the existence of this lineage in the Eocene of Southern Europe could not be excluded as well); genus *Zavaljus* was able, on the contrary, to adapt in a similar way as *Formica* Linnaeus, 1758 and *Lasius* Fabricius, 1804 [63,64] to the strongly seasonal climate possible thanks to the symbiosis: for ants with aphids [65] and for *Zavaljus* with wasps (see below). The genus *Zavaljus* remains unknown even from Baltic amber deposits yielding immeasurably more beetle fossils than Rovno amber [18]. In any case, we do not consider the Eocene distribution of *Zavaljus* boreal. Extant species clearly prefer deciduous trees (when available) to gymnosperms. The adaptation by *Zavaljus* to boreal climate may be as well a result of its symbiotic association with wasp nests, which is not common in other xenoscelines [66], and its absence in the more southern regions could hypothetically have resulted from the stronger competition there. In turn, representatives of genus *Macrophagus* collected predominantly in steppe regions as well as in forests in the south of the temperate zone, with the single Asian subtropical record from Gilan (Figure 3).

An additional explanation of the extinction of xenoscelines could be their potential competition with Asian erotylids. Distribution of these taxa towards the west was limited by the presence of the large water bodies in the Eocene time. Perhaps not coincidentally, the Oriental (and Nearctic) region are the only zoogeographic units with no available xenosceline records; the single Eastern Palaearctic xenosceline species occurs only in regions adjacent to Western Palaearctic (Figure 3). At the same time, extant Oriental and Nearctic erotylid faunas are quite diverse, and the Oriental Region appears as one of the most important diversity centers of extant erotylids. Erotylidae evolved from a microfungal diet to saprophagy (then anthophyly) or phytophagy (then feeding on dead wood) [5]. The *Macrophagus* lineage switched from a microfungal to a pollen diet (perhaps through saprophagy), and the *Zavaljus* lineage from a microfungal to a dead wood diet (perhaps through phytophagy). Leschen and Buckley [5] (p. 108) indicated: “There could have been widespread extinction in Pharaxonothinae and Xenoscelinae, resulting in scant numbers of species per genus, leaving a hypodiverse present-day remnant of a vast radiation”. Perhaps, the extinction of Xenoscelinae was caused partially by their ancient relationship with cycads [5].

Xenoscelinae is one of the stem groups of the family Erotylidae. It is possible that the younger lineages of this family (Languriinae, Cryptophilinae) forced out the older lineage into the subtropics and temperate zone: the maximum diversity of Languriinae and Cryptophilinae is in the tropical regions, in the Oriental region and the Neotropics. Cryptophilinae is not present in European amber, Languriinae is as common as Xenoscelinae (Bitterfeld amber), twice less abundant (Baltic amber) or not present (Rovno amber); on the southern coast of Subparathetys, Languriinae likely were four times less abundant than Xenoscelinae.

It appears highly likely that the comparatively warmer climate of the Rovno amber forest [67] was more favourable to xenoscelines and many other thermophile forms [53] and references therein than the Baltic one, and this is the reason for their comparatively high diversity in the Rovno coleopterofauna [37,38,42,45,52,53,57,59,68,69,70].

All in all, the present distribution of Xenoscelinae appears evidently a relic. With similar extant distribution (not counting the three Western Palearctic species) these relic taxa are habitually named “Gondwanan” (see [71,72] and references therein). Still, the domination of xenoscelines in European amber forests was so evident that the adaptations they acquired there likely contributed to the further survival of the three extant Western Palearctic genera of the subfamily.

## Data Availability

Not applicable.
